# Validation of the Writing Strategies Questionnaire in the Context of Primary Education: A Multidimensional Measurement Model

**DOI:** 10.3389/fpsyg.2021.700770

**Published:** 2021-07-05

**Authors:** Olga Arias-Gundín, Sara Real, Gert Rijlaarsdam, Paula López

**Affiliations:** ^1^Department of Psychology, Sociology and Philosophy, University of Leon, Leon, Spain; ^2^Ponferrada Associated Centre, National University of Distance Education (UNED), Leon, Spain; ^3^Research Institute for Child Development and Education, University of Amsterdam, Amsterdam, Netherlands

**Keywords:** writing strategies, questionnaire, upper-primary education, psychometrics, validity

## Abstract

Research has shown that writers seem to follow different writing strategies to juggle the high cognitive demands of writing. The use of writing strategies seems to be an important cognitive writing-related variable which has an influence on students' writing behavior during writing and, therefore, on the quality of their compositions. Several studies have tried to assess students' writing preferences toward the use of different writing strategies in University or high-school students, while research in primary education is practically non-existent. The present study, therefore, focused on the validation of the Spanish Writing Strategies Questionnaire (WSQ-SP), aimed to measure upper-primary students' preference for the use of different writing strategies, through a multidimensional model. The sample comprised 651 Spanish upper-primary students. Questionnaire data was explored by means of exploratory (EFA) and confirmatory (CFA) factor analysis. Through exploratory factor analysis four factors were identified, labeled thinking, planning, revising, and monitoring, which represent different writing strategies. The confirmatory factor analysis confirmed the adequacy of the four-factor model, with a sustainable model composed of the four factors originally identified. Based on the analysis, the final questionnaire was composed of 16 items. According to the results, the Spanish version of the Writing Strategies Questionnaire (WSQ-SP) for upper-primary students has been shown to be a valid and reliable instrument, which can be easily applied in the educational context to explore upper-primary students' writing strategies.

## Introduction

Writing has been defined as a problem-solving task that places multiple cognitive demands on the writer (Hayes, 1996). As Flower and Hayes indicated in the first cognitive model of writing (Flower and Hayes, 1980), writers have to manage several cognitively costly processes such as planning what to say, translating and transcribing those plans into written text, and revising either the plans or the written text (Alamargot and Chanquoy, 2001; Hayes, 2012). The use of these processes, especially in young writers, in whom basic transcription skills are not yet automated (Pontart et al., 2013; Alves et al., 2016; Limpo et al., 2017; Llaurado and Dockrell, 2020), consumes much of the capacity of their working memory as these processes recursively interact during composition (McCutchen, 2011).

Following a comprehensive literature review, Graham and Harris (2000) concluded that writing development seems to depend on the automation of transcription skills and the acquisition of high-levels of self-regulation in order to handle high-level processes such as planning and revision. Self-regulation, represented by the use of writing strategies, is a critical aspect of writing as it enables writers to achieve their writing goals (Zeidner et al., 2000; Santangelo et al., 2016; Puranik et al., 2019). These strategies may reduce cognitive overload as they allow writers to divide, sequence, and regulate the attention paid to the different writing processes (Kieft et al., 2006; Beauvais et al., 2011). Empirical research has shown that writers' strategic behavior during composition strongly predicts the quality of “novices” and “experts” texts (Beauvais et al., 2011; Graham et al., 2017a, 2019; Wijekumar et al., 2019). Accordingly, the use of writing strategies has been generally considered to be a critical individual writing-related variable (Kieft et al., 2008), and is a major focus of research in writing instruction (Harris et al., 2010; Graham and Harris, 2018) from the earliest stages of education (Arrimada et al., 2019). Exploring students' use of different writing strategies during composition seems to be a critical aspect and should be considered in the fields of writing and writing instructional research.

Several studies have attempted to explore how writers differ in the use of different writing strategies (Torrance et al., 1994, 1999, 2000; Biggs et al., 1999; Lavelle et al., 2002; Kieft et al., 2006, 2007, 2008). These studies identified two main writing strategies, related with the processes identified in the first seminal cognitive model of writing (Flower and Hayes, 1980), such as planning and revising. According to these studies, writers who follow a planning strategy tend to plan before beginning to write, whereas writers who prefer the revising strategy tend to plan by writing a rough draft first and then revising it. Despite the high-value of these studies, it is important to note that they only focused on analyzing the writing strategies in undergraduate (Torrance et al., 1994, 1999, 2000; Biggs et al., 1999; Lavelle et al., 2002; Arias-Gundín and Fidalgo, 2017; Robledo Ramón et al., 2018) and secondary-school students (Kieft et al., 2006, 2008). To our knowledge, just one study has explored the use of different writing strategies with upper-primary Flemish students (De Smedt et al., 2018). In this study, the authors implemented the Writing Strategies Questionnaire initially developed by Kieft et al. (2006, 2008) and identified four factors by means of exploratory and confirmatory factor analysis which were labeled thinking, planning, revising and controlling. The planning and revising strategies were consistent with those identified in previous studies with secondary school students (Kieft et al., 2006, 2008). However, in that study the authors found two additional factors. The controlling factor was defined as students' tendency to check the content or structure of their text, whereas the thinking factor make reference to the extent to which students first think about the content of their text and about their writing approach before they start writing. Thus, according to this study, it seems to be that the questionnaire assesses writing strategies in a more comprehensive way than initially intended by Kieft et al. (2006, 2008).

Additionally, it is important to consider that in all the previously reported studies, data were collected independently of the writing task through questionnaires, which may have led to biases due to self-reported estimates of writing strategies (Fidalgo and García, 2009). However, it is difficult to think of a feasible alternative for exploring writing strategies which would allow researchers to collect data from a representative sample size. Therefore, it is vitally important to conduct studies to explore the psychometric properties and the validity of these questionnaires. The advantages of exploring these aspects of the Writing Strategies Questionnaire would be the possibility of capturing students' strategy preferences non-intrusively, exploring some aspects that remain unclear about writing style (i.e., stability), and the possibility of comparing student outcomes according to their writing strategy preference in intervention studies as one key individual feature of writers at different ages (Kieft et al., 2008).

**Figure 1:**
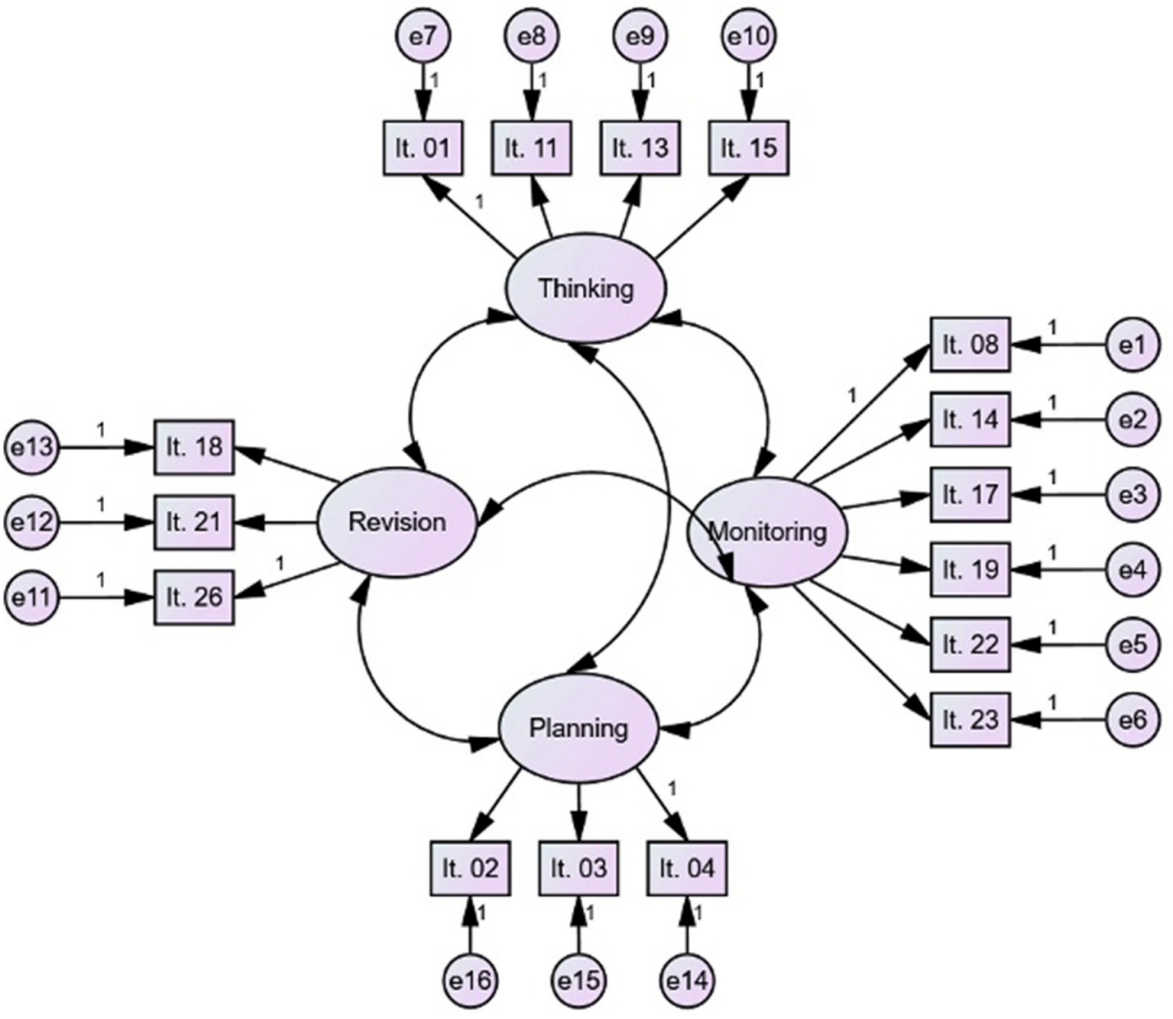
Hypothesized model of the factor structure of the WSQ-SP, composed of four interrelated factors.

Therefore, the main goal of the present study is to analyze the factor structure and validity of a Spanish version of the Writing Strategies Questionnaire (WSQ-SP) (Kieft et al., 2006, 2008) implemented with Spanish upper-primary students, analyzing the adjustment of the factorial model proposed based on the scientific literature (De Smedt et al., 2018), which consists of four interrelated factors taking into account the recursive nature of the writing process: Thinking, Planning, Revision, and Monitoring (see Figure 1). Additionally, the traditional two-factor model initially found (Kieft et al., 2006, 2008) will also be explored to test which is the most appropriate scale structure for the questionnaire.

Moreover, a second goal of the study is to analyze the factorial invariance of the proposed model by considering different variables such as gender and grade.

## Materials and Methods

### Participants

The sample comprised 651 Spanish primary school students in 16 fourth-grade (*N* = 178, 27%), 16 fifth-grade (*N* = 246; 38%), and 14 sixth-grade classes (*N* = 227; 35%). Students' ages ranged from 9 to 13 (Mage = 9.5 years, SD = 0.55 for fourth graders; Mage = 10.4 years, SD = 0.52 for fifth graders; Mage = 11.5 years, SD = 0.54 for sixth graders) and with similar proportions of boys and girls (47.19% girls in 4th grade; 48.37% girls in 5th grade; 55.07% girls in 6th grade). The students came from seven public and four semi-private schools in the city of Ponferrada, finding students from families with a high diversity of socio-economic status. However, it should be noted that most students came from families with medium to high incomes.

The criterion for choosing the participants of the study was that they should be students in 5th or 6th grade of elementary education and that Spanish should be their first language. Students in their final years of primary education were considered for developmental reasons. According to the studies of Berninger et al. (1992, 1994, 1996), planning and revision skills appear progressively during the primary education stage, with the last processes appearing in the last grades (5th and 6th). Additionally, although students with learning disabilities participated in the study, their data was not considered for the analysis. This was done on the basis of previous studies, which have shown differences in the use of high-level cognitive processes between upper-primary students with and without learning disabilities (García and Fidalgo, 2008; Graham et al., 2017b).

### Procedure

Prior to the implementation of the study, consent was requested from the Consejería de Educación de Castilla y León [Regional Department of Education of Castilla and Leon], the autonomous community in which the study was carried out. Once the study was approved by the expert committee of the regional department of Education, the researchers contacted all the schools in Ponferrada and surrounding areas. Subsequently, a meeting was held with the heads of the schools to inform them in detail about the study and the procedure to be followed during its execution. Those schools that decided to participate in the study sent the parents an information letter in which the research aims were presented, asking them for informed consent for their children to participate in the study. They were given the opportunity to express concerns and to request that their children's data not be included in the study. Following that, the study was undertaken with participation from only those students whose parents had given informed consent. The study was conducted following the Code of Ethics of the World Medical Association (Declaration of Helsinki) (Williams, 2008).

Data was collected in a natural context within regular Spanish language classes. Students were asked to complete the Spanish WSQ and writing a narrative text in a 50-min session. The questionnaire was administered by one of the researchers in this study who has a degree in Psychology and experience in administering similar kind of tests. Additionally, she received specific training on the implementation of the questionnaire. Moreover, the assessment session was audio-recorded to make sure that the assessment procedure occurred as intended.

### Measures

#### Students' Writing Strategies

In this study, we began with the 26-item questionnaire measuring students' writing strategies that has been used in previous studies (Kieft et al., 2006, 2008). Students rate their agreement with each item on a five-point scale (1–5).

For the translation of the questionnaire, we combined direct and inverse translation of the items. The questionnaire was translated from Dutch to English by a Dutch researcher who was also fluent in English and Spanish. Then this researcher and a member of the Spanish team each separately translated the English version into Spanish, in order to compare the two versions. The two Spanish translations were compared and discussed, looking for possible discrepancies.

Following that, an expert-panel assessed the suitability of the questionnaire according to the age of the target population. This panel of experts was made up of five schoolteachers with extensive experience in education (three in primary education, one in early childhood education and one in special needs education). Some changes were made to the wording to improve the understanding of the meaning of some items.

The first version of the questionnaire was then trialed with a small sample of upper-primary students to identify possible mistakes and assess general understanding. Students had no issues with it, hence no changes were made, and this produced the final version of the questionnaire (see Supplementary Material).

### Data Analysis

In order to explore the psychometric properties of the questionnaire, we first analyzed the normal distribution of each item, verifying that they gave kurtosis and skewness indices between ±7 and ±3, respectively (Kline, 2011). The magnitude and direction of the relationship between items was also analyzed using Pearson's correlation coefficient.

The validity of the factor structure was analyzed in two steps. First, we conducted exploratory factor analysis (EFA) with the aim of determining whether the items saturated the two factors of the original version or the four factors proposed in the present study (see Figure 1). Second, we performed a confirmatory factor analysis (CFA).

The maximum likelihood method was used to estimate the model using the covariance matrix of the items in order to analyze the fit of the proposed model. In order to investigate the model's goodness of fit, a number of statistics were considered: (a) absolute indices such as the Chi-square ratio and degrees of freedom (*X*^2^*/df*) and the goodness-of-fit index (GFI); (b) the comparative fit index (CFI) as an incremental fit index; (c) the adjusted goodness-of-fit index (AGFI) and the root mean square error of approximation (RMSEA) as parsimony adjustment indices. The goodness-of-fit of the model was assessed according to the following rules: (a) the *X*^2^*/df* ratio is <3; (b) values above 0.90 for the goodness-of-fit index (GFI), comparative goodness-of-fit index (CFI) and adjusted goodness-of-fit index (AGFI) are acceptable; (c) values below 0.08 for the root mean square error of approximation (RMSEA) indicate acceptable model fit (Browne and Cudeck, 1993; Hoyle, 1995; Kline, 1998; Hu and Bentler, 1999; Valdés et al., 2019).

Finally, the factorial invariance of the proposed model was analyzed by testing the fit of the model using confirmatory factor analysis (CFA) and composite reliability considering the variables gender and school year.

## Results

First, the results of the exploratory factor analysis (EFA) of the WSQ-SP are provided in order to check the factor structure of the proposed model, as well as the loading of the items on each of the factors. Second, the results of the confirmatory factor analysis (CFA) are presented showing the fit of the proposed model, as well as a comparison with the traditional two-dimensional model. Finally, the results are presented with respect to the factorial invariance of the WSQ-SP questionnaire considering gender and grade.

### Exploratory Factor Analysis (EFA)

All of the items exhibited values within the range of normal distribution (asymmetry: ranging between −1.37 and 1.20; kurtosis: raging between −0.96 and 0.82), hence the hypothesis of univariate normality was rejected in all cases (Kline, 2011).

An exploratory factor analysis (EFA) was carried out using the Maximum Likelihood extraction method and Oblimin rotation. The data showed a good fit for this kind of model, evidenced by Bartlett's sphericity test (χ^2^ (171) = 2216.68, *p* < 0.000) and the Kaiser-Meyer-Olkin (KMO) value of 0.83 (Lloret-Segura et al., 2014). As a criterion for item inclusion, factor weights >0.30 were considered for only one of the factors, reflecting the theoretical soundness of the scale (Hair et al., 1999). Ten items were excluded because they did not match the different factors (items 5, 6, 7, 9, 10, 12, 15, 16, 20, and 24). The results showed that the 16 items of the scale are grouped into four factors which were theoretically identified and retained. These factors were labeled revising, monitoring, thinking, and planning and together explain 32.08% of the variance. The first factor, *monitoring*, corresponds to how much students checked the content or structure of their text during composition. This factor consisted of six items explaining 18.0% of the variance and had a composite reliability of 0.82. The second factor, *revising*, is related to how much students revised the content of their text once the text was written. This factor included three items explaining 7.9% of the variance and had a composite reliability of 0.85. The third factor, *planning*, is related to how much students thought about the content of their text in advance, using external planning devices such as a draft sheet. This factor included three items explaining 3.8% of the variance and had a composite reliability of 0.75. Finally, the fourth factor, *thinking*, corresponds to how much students needed to have a clear idea of the content or structure of the text in their minds before they started to write. This factor consisted of four items explaining 2.4% of the variance and a composite reliability of 0.79 (see Table 1).

**Table 1:** Exploratory factor analysis (EFA) of the WSQ-SP.

**Items**	**Factor loadings**				**Commonality**
	**F1**	**F2**	**F3**	**F4**	
8. While writing, I regularly check whether my text doesn't contain sentences that are too long or incorrect.	0.50				0.29
^*R*^14. When writing, I sometimes write paragraphs of which I know that they are not yet correct, but I prefer to continue writing.	0.38				0.18
^*R*^17. I usually hand in my text without checking whether the paragraphs are well arranged.	0.70				0.46
19. Before I hand in my text, I check whether it is structured logically.	0.70				0.58
^*R*^22. When I reread my texts, sometimes they are very chaotic.	0.33				0.12
23. I have to reread the texts I wrote, to prevent redundancies.	0.33				0.27
18. When I reread and rewrite my text, the structure of my text changes a lot.		0.71			0.51
21. When I rewrite my texts, the content often changes a lot.		0.73			0.50
26. When I finished writing, I reread and improve a lot: there might change a lot in my text.		0.56			0.40
2. I always use a diagram before I start to write.			−0.39		0.16
3. Before writing a text, I jot down some notes on a scribbling paper. Later, I elaborate these notes.			−0.83		0.62
4. Before I start to write a text, I prefer to write down some thoughts on a scribbling paper to discover what I think about the topic.			0.65		0.43
1. When I write a text, I spend a lot of time thinking on how to approach it.				0.33	0.19
11. I need to have my thoughts clear, before I can start to write.				0.60	0.32
13. Before I write down a sentence, I have it clear in my mind.				0.41	0.19
15. Writing helps me to clarify my thoughts.				0.65	0.17
				Correlations	
Monitoring	-				
Revision	0.03	-			
Planning	0.27^*^	0.21^*^	-		
Thinking	0.45^*^	0.22^*^	0.27^*^	-	

* *p < 0.01*.

R *Items recoded in the analyses*.

### Confirmatory Factor Analysis (CFA)

We performed CFA for the 16 items in the WSQ-SP using Amos software in SPSS. We used Maximum Likelihood (ML) factor analysis with the CFA command. The results of the CFA suggest that overall, the model had a good fit to the data according to the indices (χ^2^/*df* = 2.23; GFI = 0.96; AGFI = 0.95; CFI = 0.93; RMSEA = 0.04 CI (0.03–0.05).

**Figure 2:**
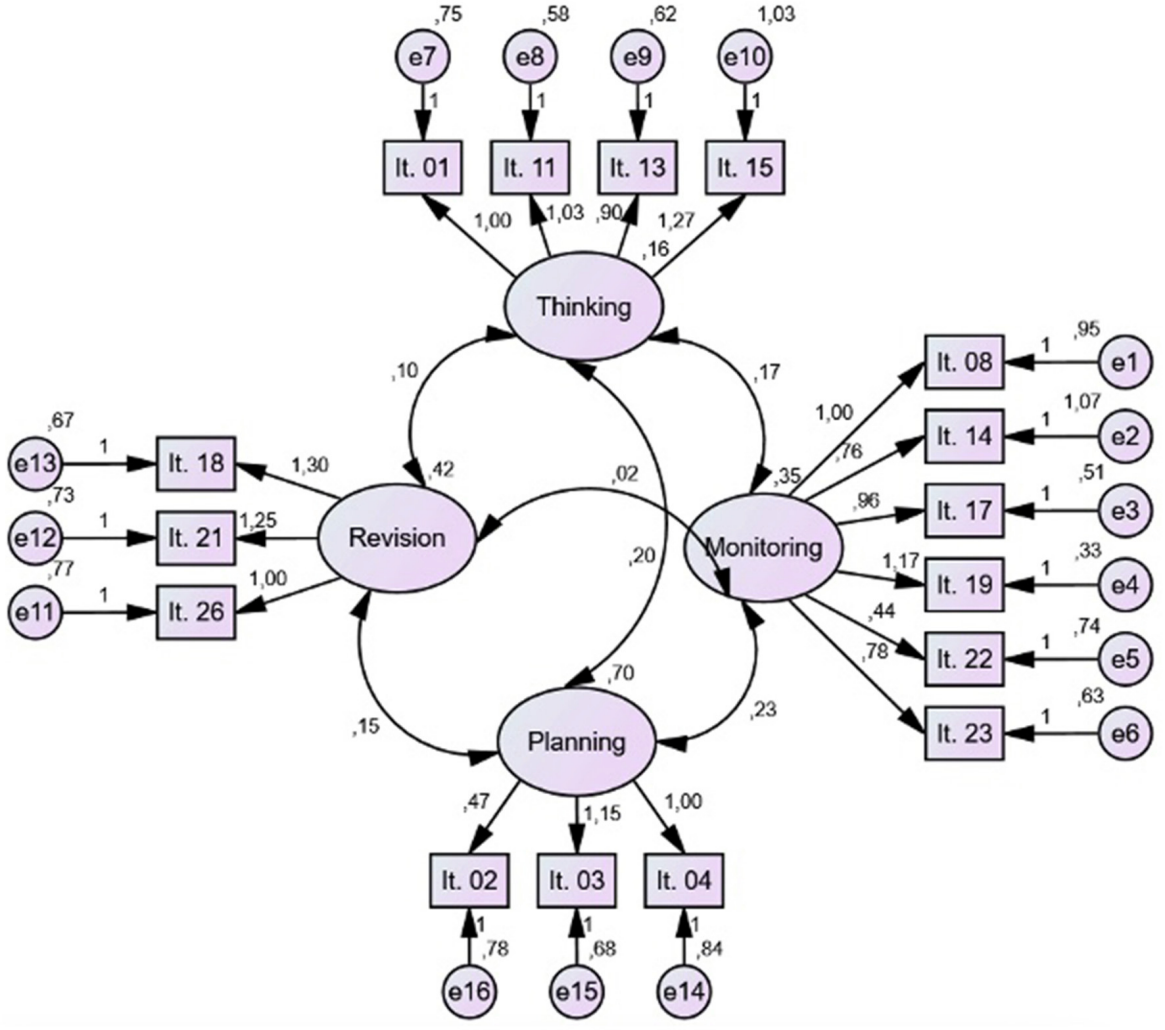
Path diagram of the hypothesized model. Confirmatory Factor Analysis of the questionnaire.

The values of the regression coefficients suggest that the factors explained an acceptable part of the variance of the items (see Figure 2). The correlation between the factors indicated that the factors were related but did not present problems of collinearity.

Considering that the proposed model was corroborated by the results, it was compared with the traditional two-dimensional structure identified in previous studies (Kieft et al., 2006, 2008). The model proposed in this study exhibited the best factorial fit (see Table 2).

**Table 2:** Goodness of fit indices for each model of the CFA of the WSQ-SP (*N* = 651).

**Model**	***X^**2**^/df***	***GFI***	***AGFI***	***CFI***	***RMSEA* (IC al 90%)**
4 Factors^*^	2.23	0.96	0.95	0.93	0.04 (0.03–0.05)
2 Factors^**^	6.83	0.86	0.81	0.65	0.09 (0.08–0.10)

* *Proposed model with 4 factors: Monitoring, Revision, Planning, and Thinking*.

** *Traditional two-dimensional model: Planning and Revision*.

### Factor Invariance Analysis

To check that the effectiveness of the model was not significantly affected by the features of the sample, the proposed model was subjected to CFA by selecting the sample based on gender and grade. These two variables were chosen for the following reasons. Gender was considered because some studies have shown it to be a variable that can influence student learning and achievement in general (e.g., Reilly et al., 2019) and specifically in the use of cognitive writing strategies (e.g., Berninger et al., 1992; Jones, 2011). Additionally, grade was chosen because it is during this period of schooling that higher-level cognitive processes related to textual planning and revision appear following different rates of development (Berninger et al., 1992, 1994, 1996). The aim was to ensure that the questionnaire is reliable regardless of gender or grade.

As Table 3 shows, the composite reliability of each factor in all of the proposed models, based on the characteristics of the sample and their combinations, is high (ranging between: 0.81 and 0.92 for the monitoring factor; 0.70 and 0.93 for the thinking factor; 0.70 and 0.82 for the planning factor; and 0.81 and 0.91 for the revising factor). The model shows a good overall fit for the gender and grade variables, with the indicators meeting the established parameters. There was just one exception for the adjusted goodness-of-fit index (AGFI) in the case of 4th grade students (0.88), which was very close to the desired value (0.90). When the model was analyzed based on the interaction of gender and grade, the absolute index, Chi-square ratio and degrees of freedom, and the RMSEA as the parsimony adjustment index, demonstrated acceptable model fit, with the remaining indicators being close to the desired value (0.90). However, it is important to note that when the model was analyzed based on gender-grade interaction, the sample shrank considerably. This influenced the results, given that CFA is sensitive to sample size. The literature recommends performing CFA analysis with samples of more than 200 participants (Valdés et al., 2019). In all of the cases analyzing the model with samples smaller than 200 students, some indicators did not give the desired values, as Table 3 shows.

**Table 3:** Goodness-of-Fit Indices for the proposed model of the questionnaire based on sample features.

**Model (n)**	***X^**2**^***	**p**	***X^**2**^*/*df***	**GFI**	**AGFI**	**CFI**	**RMSEA (IC al 90%)**	**Composite reliability**			
								**Monitoring**	**Thinking**	**Planning**	**Revising**
**Gender**											
Girls (*n* = 328)	176.92	0.000	1.80	0.94	0.91	0.90	0.05 (0.04–0.06)	0.86	0.76	0.72	0.88
Boys (*n* = 323)	150.64	0.001	1.54	0.94	0.92	0.93	0.04 (0.03–0.06)	0.86	0.84	0.80	0.86
**Grade**											
4th (*n* = 178)	147.36	0.001	1.50	0.91	0.88	0.91	0.05 (0.03–0.07)	0.86	0.85	0.74	0.81
5th (*n* = 246)	151.01	0.000	1.54	0.93	0.90	0.91	0.05 (0.03–0.06)	0.87	0.73	0.77	0.86
6th (*n* = 227)	118.67	0.076	1.21	0.94	0.92	0.97	0.03 (0.00–0.05)	0.86	0.74	0.76	0.89
**Gender and Grade**											
4th Girls (*n* = 82)	117.23	0.090	1.19	0.85	0.80	0.91	0.05 (0.00–0.08)	0.81	0.74	0.76	0.89
5th Girls (*n* = 119)	136.63	0.006	1.39	0.88	0.83	0.89	0.06 (0.03–0.08)	0.85	0.70	0.79	0.87
6th Girls (*n* = 125)	146.16	0.001	1.49	0.88	0.83	0.85	0.06 (0.04–0.08)	0.92	0.85	0.73	0.91
4th Boys (*n* = 93)	111.56	0.165	1.14	0.88	0.84	0.95	0.04 (0.00–0.07)	0.86	0.93	0.79	0.83
5th Boys (*n* = 97)	161.09	0.000	1.64	0.83	0.78	0.73	0.08 (0.06–0.10)	0.86	0.85	0.70	0.84
6th Boys (*n* = 100)	102.62	0.355	1.05	0.90	0.86	0.99	0.02 (0.00–0.06)	0.86	0.71	0.82	0.90

## Discussion

The main goal of the present study was to analyze the factor structure and validity of the Spanish WSQ-SP with upper-primary students. An additional goal was to analyze the factorial invariance of the proposed model by considering different variables such as gender and grade.

With regard to the first goal of the study, the results relating to the questionnaire's factor structure were in line with the previous study carried out with Flemish upper-primary students (De Smedt et al., 2018) in which four factors were identified; planning, revising, monitoring and thinking. In addition, on comparing this model with the two-factor model (i.e., planning and revising), generally identified in previous studies with more expert writers (Kieft et al., 2006, 2008), the four-factor model demonstrated a better match with the questionnaire structure.

This four-factor model is consistent with the differentiation of planning and revision processes that have generally been considered in terms of their occurrence during the process of writing a text (Berninger et al., 1994). As planning and revision can occur before or during translating, a distinction was made between advanced and online planning, post-translation and online revision. In this way, the *thinking* and *planning* factors were related to the two different, but complementary, ways of planning. According to previous studies, writers differ in how they plan. While some writers make an outline in note form before drafting, others plan without producing an outline. This latter form of planning has been called “mental planning” (Kellogg, 1988; Torrance et al., 2000). Thus, the *thinking* factor would correspond to mental planning while the *planning* factor would correspond to outline planning. Similarly, the *revising* and *monitoring* factors can be interpreted according to when revision occurs. According to Berninger and Swanson (1994) considering the timing of revision it is possible to differentiate between online revision (i.e., revision that takes place during composition) and post-translation revision (i.e., revision that takes place after composition). Thus, the revising factor would correspond with post-translation revision while the monitoring factor would correspond with online revision. In other words, the results of the present study indicate that the questionnaire is not only exploring students' use of planning and revising strategies in a general way, but rather also assessing different types of planning and revision strategies depending on when they take place when students are writing a text. These results are in line with the arguments presented by Kieft et al. (2007) and Tillema et al. (2011), who pointed out that the revising scale was composed not only of items related to post-translation revision but also to monitoring. Moreover, the better fit of the four-factor model can be explained based on the fact that these processes seem to have different rates of development (Berninger et al., 1992, 1994, 1996). Based on the implementation of cross-sectional studies with students aged between 6 and 15 years old, the authors found that online planning and revision seems to appear at around ages 6–9 (1st−3rd grades). The authors also found that advanced planning and post-translation revision were the last processes to appear around the last years of primary school (ages 9–12; 4th−6th grades). This would clearly explain why the four-factor model has a better fit to the data from primary school pupils. Here, it is also important to consider that the four factors were shown to exhibit correlation but no problems of collinearity were found. This result is in line the view of writing as a recursive activity in which one process may interrupt others during composition (Flower and Hayes, 1980).

In terms of the second goal of the study, analyzing the factorial invariance of the proposed model by considering different variables such as gender and grade, the results showed that the questionnaire structure was independent of the feature of the sample. The results of the present study seem to be generalizable to upper-primary students regardless of gender or grade.

In summary, the major contribution of this study is the validation of the WSQ-SP with upper-primary students, as validation is a critical step for the development of reliable measurement tools in all scientific domains (Muñiz and Fonseca-Pedrero, 2019). From this study, we can conclude that the questionnaire provides more precise information than initially expected and it is a suitable tool for easily, reliably assessing upper-primary students' writing.

The validation of this questionnaire is a first step toward a reliable analysis of this variable, which will continue with analyzing aspects that have not yet been investigated, such as its stability, the moderating effect it has on writing intervention in upper-primary students (Kieft et al., 2006, 2008), and the effect of instruction itself on writing. Having a validated questionnaire will also make it possible to analyze the relationship between students' use of strategies and other important writing-related variables such as reading (Fidalgo et al., 2014; Qin and Liu, 2021), motivation (Rocha et al., 2019), and students' knowledge (Wijekumar et al., 2019). It would also be interesting to analyze the relationship between the results provided by this scale and the writing processes students follow through the use of online measures such as the triple task (García and Fidalgo, 2008; Fidalgo et al., 2014) and thinking aloud (López et al., 2019).

Finally, as an educational contribution, this instrument may be a useful tool that will help provide teachers with information about their students' strategies and consequently help them to adapt the writing instruction according to their needs. All of this, without a doubt, will have a positive impact on students' writing performance, not only in initial educational levels (e.g., López et al., 2017), but also in later educational stages, such as at University, where students often find it difficult to write academic texts (Connelly et al., 2005).

## Data Availability Statement

The raw data supporting the conclusions of this article will be made available by the authors, under request to the corresponding author, without undue reservation.

## Ethics Statement

The studies involving human participants were reviewed and approved by Consejería de Educación de Castilla y León. Written informed consent to participate in this study was provided by the participants' legal guardian/next of kin.

## Author Contributions

All authors listed have made a substantial, direct and intellectual contribution to the design of the work, analysis and data interpretation, drafting and revising it critically, and approved it for publication.

## Conflict of Interest

The authors declare that the research was conducted in the absence of any commercial or financial relationships that could be construed as a potential conflict of interest.
