# Supplementary material for: Validation of the Writing Strategies Questionnaire in the Context of Primary Education: A Multidimensional Measurement Model
Source: Front Psychol. 2021 Jul 5;12:700770. doi: 10.3389/fpsyg.2021.700770 (PMC8287024; doi:10.3389/fpsyg.2021.700770)
Supplement: Supplementary file 1 [file Data_Sheet_1.docx]

Supplementary Material

Validation of the writing strategies questionnaire in the context of primary education: A multidimensional measurement model

Olga Arias-Gundín^1^, Sara Real^2^, Gert Rijlaarsdam^3^, Paula López^1*^

*** Correspondence:** plopg@unileon.es

# Supplementary material: Writing Strategies Questionnaire in Spanish (WSQ-SP)

### Nombre y apellidos:

A continuación, vas a leer una serie de frases sobre distintas actividades relacionadas con la escritura de un texto.

Debes leer cada frase y señalar en qué medida realizas la actividad que se indica en ella.

Para contestar debes elegir un número del 1 al 5, en función de si:

| 1 - Nunca lo haces. |
| --- |
| 2 - Casi nunca lo haces. |
| 3 - Unas veces sí lo haces y otras no. |
| 4 - Casi siempre lo haces. |
| 5 - Siempre lo haces. |

Ten en cuenta que tus respuestas nunca van a ser incorrectas. Las frases que leerás tan solo reflejan diferentes actividades que cada persona puede utilizar o no para escribir un texto.

**¡Es muy importante que seas sincero y respondas lo que realmente haces o piensas cuando escribes!**

*¡Vamos a ver un ejemplo!*

|  | 1  Nunca | 2  Casi nunca | 3  Unas veces sí, otras no | 4  Casi Siempre | 5  Siempre |
| --- | --- | --- | --- | --- | --- |
| Ejemplo:  *Antes de escribir un texto pienso ideas* |  |  |  |  |  |

*Si antes de escribir un texto NUNCA piensas ideas... deberás marcar una cruz en el 1.*

*Si CASI NUNCA piensas ideas antes de escribir un texto... deberás marcar una cruz en el 2.*

*Si UNAS VECES SÍ piensas ideas y OTRAS NO... marcarás un 3.*

*Si CASI SIEMPRE piensas ideas antes de escribir un texto... marcarás con una cruz en el 4.*

*Si SIEMPRE piensas ideas antes de escribir un texto.... marcarás con una cruz el 5.*

*¿Lo habéis entendido? ¡Pues vamos a comenzar!*

|  | 1  Nunca | 2  Casi nunca | 3  Unas veces sí, otras no | 4  Casi Siempre | 5 Siempre |
| --- | --- | --- | --- | --- | --- |
| 1. Cuando escribo un texto, dedico mucho tiempo a pensar cómo voy a hacerlo. |  |  |  |  |  |
| 1. Antes de comenzar a escribir hago un esquema. |  |  |  |  |  |
| 1. Antes de escribir un texto, apunto algunas ideas en una hoja en sucio y luego las explico mejor en mi texto. |  |  |  |  |  |
| 1. Antes de comenzar mi texto, escribo algunas de mis ideas en una hoja en sucio, para darme cuenta de lo que se sobre el tema. |  |  |  |  |  |
| 1. Pensar en qué y cómo voy a escribir mi texto no es útil para mí. |  |  |  |  |  |
| 1. Cuando comienzo a escribir no sé cuál va a ser la información de mi texto. |  |  |  |  |  |
| 1. Antes de comenzar a escribir tengo claro qué quiero conseguir en la persona que lee mi texto. |  |  |  |  |  |
| 1. Mientras estoy escribiendo compruebo cada poco que mi texto no tenga frases incorrectas o demasiado largas. |  |  |  |  |  |
| 1. Cuando sé en general qué voy a escribir, escribo mi texto con mucha facilidad. |  |  |  |  |  |
| 1. Cuando escribo un texto, es difícil para mí tener ideas sobre las que escribir. |  |  |  |  |  |
| 1. Necesito tener mis ideas claras antes de comenzar a escribir. |  |  |  |  |  |
| 1. Cuando escribo un texto, de vez en cuando pienso si lo entenderán las personas que lo lean. |  |  |  |  |  |
| 1. Antes de escribir una frase la tengo clara en mi cabeza. |  |  |  |  |  |
| 1. A veces escribo párrafos que sé que aún no están totalmente bien, pero prefiero seguir escribiendo |  |  |  |  |  |
| 1. Escribir me ayuda a tener más claro lo que pienso. |  |  |  |  |  |
| 1. Los textos que escribo no son muy originales. |  |  |  |  |  |
| 1. Entrego mi texto sin comprobar que los párrafos estén bien organizados. |  |  |  |  |  |
| 1. Cuando vuelvo a leer y modifico mi texto, su organización cambia mucho. |  |  |  |  |  |
| 1. Antes de entregar mi texto, compruebo si está correctamente organizado. |  |  |  |  |  |
| 1. No le doy importancia a si escribo las frases de forma incompleta o sin alguna idea. |  |  |  |  |  |
| 1. Cuando modifico mis textos, la información cambia mucho. |  |  |  |  |  |
| 1. Cuando vuelvo a leer mis textos son muy liosos. |  |  |  |  |  |
| 1. Vuelvo a leer los textos que escribo para evitar repetir la misma información varias veces. |  |  |  |  |  |
| 1. No doy importancia a si estoy contento con mi texto. |  |  |  |  |  |
| 1. Antes de comenzar a escribir, necesito saber cuál será la información de mi texto. Por lo tanto, pensar qué y cómo escribir es importante. |  |  |  |  |  |
| 1. Cuando acabo de escribir vuelvo a leer y mejoro mucho mi texto, haciendo muchos cambios. |  |  |  |  |  |
